# Repurposing metformin as a potential anticancer agent using *in silico* technique

**DOI:** 10.1007/s40199-024-00523-0

**Published:** 2024-06-26

**Authors:** Mona Mahfauz, Ozel Yuruker, Rasime Kalkan

**Affiliations:** 1 Faculty of Pharmacy, Cyprus Health and Social Sciences University, Güzelyurt, Cyprus; 2 Faculty of Medicine, European University of Lefke, Mersin 10, Lefke, 99728 Northern Cyprus Turkey

**Keywords:** Metformin, Drug repurposing, Cancer, In-silico

## Abstract

**Background:**

The focus on repurposing readily available, well-known drugs for new, creative uses has grown recently. One such medication is metformin, a drug commonly used to manage diabetes, which shows a favorable correlation between its use and lower cancer morbidity and death. Numerous investigations and clinical trials have been conducted to evaluate the possible application of metformin as an anticancer medication in light of this conclusion.

**Objective:**

This study used 'pathway/gene-set analysis' Gene2drug, a resource for Gene Ontology (GO), and DepMap to determine whether metformin would be potentially advantageous for treating cancer.

**Methods:**

A total of 1826 tumor cell lines were analyzed using the Drug Sensitivity (Primary Purposing Primary Screening) 19Q4 Tool.

**Results:**

9 genes from 402 genes, *SGPL1*, *CXCR6*, *ATXN2L*, *LAMP3, RTN3, BTN2A1, FOXM1, NQO1,* and *L1TD1* in 1826 cancer cell line showed statistical sensitivity to metformin.

**Conclusion:**

This *in-silico* study showed the sensitivity of specific cancer cell lines to metformin. Therefore, holding promises for metformin and tumor-targeted treatment strategies. It is recommended, however, to conduct further research into its potential effectiveness and mechanism of action.

**Graphical Abstract:**

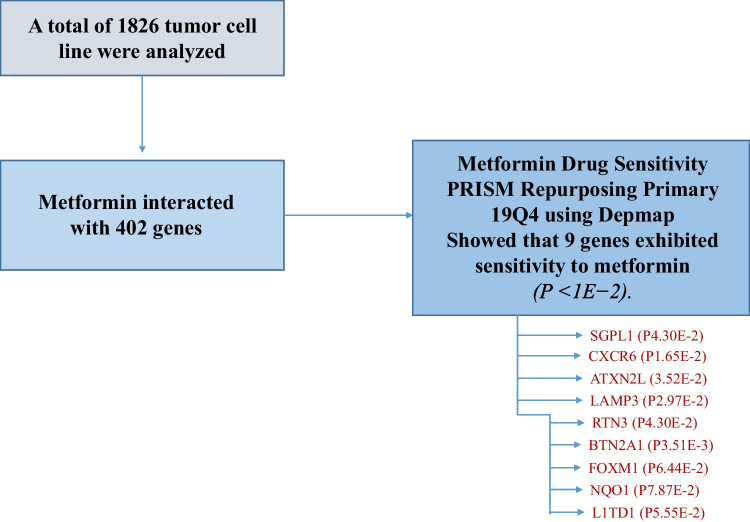

**Supplementary Information:**

The online version contains supplementary material available at 10.1007/s40199-024-00523-0.

## Introduction

Cancer is one of the global health problems [1] and according to GLOBOCAN 2020, there were 10.3 million cancer-related deaths and 19.3 million new cases of the disease worldwide despite medical advancement [2]. Currently, cancer is typically treated with a combination of medicines to extend the lifetime or treat the disease. Surgery, radiation, chemotherapy, hormone therapy, biological therapy, and targeted therapy are among the therapeutic options available for cancer patients [3]. Yet, in recent years, the knowledge of the processes by which cancer cells develop chemo-resistant properties has exploded. These cells may adopt a common (multidrug) or drug-specific (particular drug) resistant phenotype by following a variety of genetic modification pathways, either naturally (intrinsic resistance) or as a result of chemotherapy (acquired resistance)[4]. On the other hand, the process of finding and developing new drugs is expensive and time-consuming. Typically, it takes many years to develop a molecule to the point where it can be used in medicine. Newly discovered medications are not always associated with greater therapeutic benefits[5]. Drug repositioning, for instance, also known as drug repurposing, is the practice of using already-approved medications for new indications or to address medical needs with drugs that have been clinically authorized to be safe, and the dose range and formulation have already undergone extensive research. Therefore, drug development through medication repositioning may have a reduced risk of failure, and take less time to complete preclinical and phase I/II clinical trials by taking advantage of the wealth of information about clinically authorized pharmaceuticals[6, 7]. One of the oldest and most extensively studied drugs is metformin, which is the initial line of treatment for type 2 diabetes[8]. Metformin, a derivative of biguanide for type 2 diabetes, is the recommended treatment due to its effectiveness, affordability, and long history of use which has received FDA(Food and Drug Administration) approval in 1994[9]. It is used alone or in several combination products with other antidiabetic medications. Because it is well-researched and has been used for many years, metformin is the recommended treatment for type 2 diabetes. Additionally, there are several non-FDA-approved uses for metformin, including the control of weight gain brought on by antipsychotics, the prevention of type 2 diabetes, and the treatment and prevention of polycystic ovarian syndrome (PCOS). In addition, the only antidiabetic for pre-diabetes that the American Diabetes Association currently approves is metformin. Metformin is being investigated for its potential antiaging, anticancer, and neuroprotective properties[10] where researchers are investigating whether metformin can lower the risk of cancer in individuals with type 2 diabetes. These consist of prostate, colon, and breast malignancies. In addition, it lowered the chances of stroke and dementia. Studies have shown that individuals with diabetes who take metformin have reduced rates of stroke, dementia, and cognitive impairment when compared to those who do not[11]. In summary, metformin's multifaceted benefits extend beyond glycemic control, encompassing applications in prediabetes, gestational diabetes, and ongoing investigations into cancer risk reduction, cognitive health, and potential anti-aging effects. The off-label use of metformin underscores its versatility and potential impact on various aspects of health beyond diabetes management. On the other hand, metformin use has been determined to be generally safe, with the most frequent side effects being moderate gastrointestinal discomfort[12]. Yet, less than 1 in 10,000 persons experience serious adverse effects. For this reason, metformin is crucially advised to be undertaken solely under the supervision and guidance of a healthcare professional[13]. Apart from this, according to numerous retrospective studies, Metformin was found to possess some anticancer activity, plus its use was connected to a reduction in both cancer-related and all-cause mortality, based on a meta-analysis of data from cohort and observational studies [14]. Metformin demonstrates a growing body of evidence supporting its ability to impede the growth, survival, and metastasis of diverse tumor cell types, spanning breast, liver, bone, pancreas, endometrial, colorectal, kidney, and lung cancers [10]. Its anti-cancer effects are attributed to both direct and indirect regulation of cellular metabolism. Through AMP-activated protein kinase (AMPK) dependent and -independent pathways, metformin exerts direct effects. Specifically, metformin activates AMPK, leading to the inhibition of the mammalian target of rapamycin (mTOR) signaling. Consequently, disruptions in protein synthesis occur, suppressing cell growth and proliferation. Notably, metformin may interfere with the crosstalk between G protein-coupled receptors (GPCRs) and insulin receptor signaling systems, potentially contributing to the inhibition of pancreatic cancer proliferation [15]. In several tumor models, the modulation of tumor-infiltrated effector immune cells, including CD8 + , CD4 + T cells, and natural killer (NK) cells, as well as suppressor immune cells such as T regulatory cells, tumor-associated macrophages (TAMs), and myeloid-derived suppressor cells (MDSCs), can be achieved by metformin. The role of metformin in modulating tumor-infiltrating immune cells in various preclinical models and clinical trials was discussed in a review study where both preclinical and clinical studies indicate that promise is held by metformin as adjunctive therapy in cancer treatment through the modulation of the immune response within the tumor microenvironment [16]. A further review study has demonstrated that in addition to altering the tumor microenvironment to stop the growth, survival, and spread of tumor cells, metformin also suppresses the formation of cancer. The underlying molecular pathways include reduced reactive oxygen species (ROS) generation, Deoxyribonucleic acid (DNA) damage, autophagy and apoptosis, activation of p53, suppression of the mTOR signaling, and inflammatory response. Metformin has also been demonstrated to have positive benefits on age-related diseases, renal diseases, obesity, cardiovascular diseases, and liver disorders, all of which lower the chance of death [17]. Although numerous studies have demonstrated the potential effect, only a limited number have concentrated on a singular type of cancer while investigating the specific biological targets of metformin at the genetic level. This study aimed to evaluate metformin's effectiveness in 1826 different cancer cell lines as a potential anticancer treatment drug. The advantage of drug repurposing is to confirm metformin’s potential anti-cancer effects in 1826 cell lines, which means screening the large number of cell lines. This in silico study helps us to shorten time frame cycles, needs less investment, and prevents failure in later stages of research.

## Materials and methods

Scientific experimentation is revolutionized by in-silico approaches, which substitute computer simulations for traditional lab work. The computer turns into an advanced virtual lab that replicates interactions between genes and specific diseases in biology. Genes are examined utilizing in-silico techniques, which are similar to a digital library search to identify important genes associated with disorders. Genes are conceived as the instruction manuals for the human body. Real experiments are guided by predictions derived from computer models, which expedites the research process. In silico simulations evaluate the interactions between chemicals and our biology in drug discovery, providing a time and money-efficient method. The techniques are useful for personalized medicine because they can forecast the best course of action based on a patient's specific genetic composition. In the end, in-silico methods advance our knowledge of health and illness in a way that is comparable to exploring a virtual environment to learn about the complexities of our bodies [18].

### Data source

DSEA, Drug Set Enrichment Analysis was used to be able to find relevant gene targets for the metformin drug. The in-silico screening was conducted by the mean of Gene2drug [19–21, 44]. Based on PRISM viability assays on 1826 cancer cell lines (Shown in Suppl 1), DepMap data were analyzed for the antitumor activities of metformin and its target genes[19]. Metformin Drug Sensitivity PRISM Repurposing Primary 19Q4 and Damaging Mutation in Depmap were used to sort cancer cells for sensitivity to metformin[19, 20]. The gene expression profiles were downloaded from the Cancer Cell Line Encyclopedia (Expression 22Q2 Public) [21].

### Analysis of gene-drug interactions

Damaging mutation and Drug Sensitivity (Primary Purposing Primary Screening) 19Q4 were used to sort cancer cell lines for sensitivity to Metformin [19]. A list of genes was generated, ranked by P-value, and decided the output of Gene2Drug. Genes that had a P value lower than 1E-2 were included.

### Validating the antitumor activity of metformin

The sensitivity of metformin was extracted from DepMap [19].

## Results

### In-silico screening of potential genes

In total 1826 cancer cell lines were analyzed (Suppl.1).

### The sensitivities of cancer cells to metformin

The sensitivities of cancer cells to metformin drugs were extracted from PRISM. The interaction of metformin with genes (402 genes) was demonstrated by PRISM Repurposing Primary 19Q4 and AUC Drug sensitivity (area under the drug concentration) by CTD^2 (Suppl 2) and evaluated by using DepMap. Genes exhibiting a significant (P < 1E − 2) were selected to discover whether cells were sensitive to metformin. Metformin Drug Sensitivity PRISM Repurposing Primary 19Q4 using Depmap showed 9 genes exhibiting sensitivity to metformin, Sphingosine-1-phosphate lyase, *SGPL1* (P4.30E-2), C-X-C motif chemokine receptor 6, *CXCR6* (P1.65E-2), Ataxin-2-like, *ATXN2L* (3.52E-2), Lysosome-associated membrane glycoprotein 3, *LAMP3* (P2.97E-2), reticulon 3, *RTN3* (P4.30E-2), Butyrophilin Subfamily 2 Member A1, *BTN2A1* (P3.51E-3), Forkhead Box M1, *FOXM1* (P6.44E-2), NAD(P)H Quinone Dehydrogenase 1, *NQO1* (P7.87E-2) and Lineage-specific 1 transmembrane domain-containing protein *L1TD1* (P5.55E-2).. The significant genes list and relevant cell lines have been listed in Table 1.

**Table 1:** The list of genes and associated cancer cell lines

Gene	Pearson	Spearman	Slope	Intercept	p-value (linregress)	Cell Lines
SGPL1	-0.087	-0.070	-1.03E-2	3.09E-3	4.30E-2	Bladder Uroepithelial Carcinoma
CXCR6	-0.102	-0.072	-1.22E-2	3.33E-3	1.65E-2	Colorectal Adenocarcinoma
ATXN2L	-0.090	-0.075	-4.46E-2	3.84E-2	3.52E-2	Endometrial Carcinoma Colorectal Adenocarcinoma Overian Epithelial tumor MelanomaPacreatic adenocarcinoma Urethral cancer Eusophagogastric adenocarcinoma
LAMP3	-0.093	-0.052	-1.56E-2	5.58E-3	2.97E-2	Endometrial Carcinoma Colorectal Adenocarcinoma
RTN3	-0.122	-0.094	-2.04E-2	6.18E-3	4.30E-3	Endometrial carcinoma Diffuse glioma
BTN2A1	-0.125	-0.107	-4.87E-2	2.25E-2	3.51E-3	Diffuse glioma Hepatocellular carcinoma Colorectal Adenocarcinoma Overian epithelial tumor Esophagogastric adenocarcinoma Colorectal Adenocarcinoma
FOXM1	-0.079	-0.049	-2.09E-2	1.17E-2	6.44E-2	Endometrial carcinoma Overian epithelial tumor Colorectal Adenocarcinoma Bladder urothelial carcinoma
NQO1	-0.075	-0.086	-1.99E-2	7.94E-3	7.87E-2	Endometrial carcinoma Melanoma
L1TD1	0.082	0.079	1.37E-2	1.96E-3	5.55E-2	Non-small cell lung cancer Esophagogastic carcinoma

## Discussion

A total of 1826 cell lines as a possible target for metformin were suggested in our in-silico screening and 9 genes showed sensitivity to metformin. *SGPL1* (Sphingosine-1-Phosphate Lyase 1) gene allows for sphinganine-1-phosphate aldolase activity. It is involved in the sphingolipid and fatty acid metabolism as well as apoptotic signaling pathways. The development of cancer cells, their directed chemo-attraction, and the promotion of chemo-resistance mechanisms have all been linked to high sphingosine-1-phosphate (S1P) concentrations and defects in S1P breakdown. Through its irreversible S1P degradation activity, the endoplasmic reticulum (ER) membrane-localized enzyme sphingosine-1-phosphate lyase (SGPL1) plays a critical part in preventing S1P overstimulation in tumor cells [22]. Therefore, transformation and carcinogenesis are prevented by SGPL [23]. A study assessing the effects of Metformin as an add-on therapy against glioblastoma proved that metformin promotes the expression of genes encoding the enzymes that catabolize S1P into sphingosine. Supporting the sensitivity of *SGPL 1* gene to metformin in our study. *CXCR6* or C-X-C Motif Chemokine Receptor 6 gene is a Protein Coding gene. The encoded protein and its unique ligand, chemokine ligand 16 (CCL16), are a component of a signaling pathway that controls the migration of T lymphocytes to different peripheral tissues, including the liver, spleen red pulp, intestine, lungs, and skin, and fosters cell–cell contact with dendritic cells and fibroblastic reticular cells. Additionally, CXCR6/CCL16 regulates resident memory T cell localization to various lung compartments and maintains resident memory T lymphocytes in the airways, which serve as an essential initial line of defense against respiratory infections [24]. It has been evident in recent years that chemokine and its receptors play a role in either promoting or inhibiting the growth of tumors [25]. A paper conducted to evaluate the expression of the chemokine receptor, *CXCR6* in human colorectal adenocarcinomas showed that *CXCR6* can increase the number of antigen-presenting cells in the mucosa and the down-regulation of CXCR6 found in tumor tissue may be a tactic used by tumors to avoid conflict with leukocytes that have infiltrated the tumor, such as antigen-presenting cells. The ATXN2L gene encodes a protein related to ataxin-2, although its specific function remains unidentified. The spinocerebellar ataxia (SCAs) protein family, which includes this protein, is linked to a wide range of neurodegenerative diseases. For this gene, many alternatively spliced transcripts encoding various isoforms have been discovered [26]. In tumor tissue, *ATXN2L* was markedly overexpressed, and the frequency of this overexpression rose as the cancer's stage advanced. Moreover, this overexpression can also contribute to the resistance to chemotherapeutic agents [27]. *LAMP3* (Lysosomal Associated Membrane Protein 3) is a protein-coding gene. The LAMPs family is crucial to the process of autolysosome fusion. Recent research has shown that autophagy controls hepatic lipolysis. However, it is unclear what role LAMP3 plays physiologically in lipid metabolism [28]. According to studies, *LAMP3* is overexpressed in a variety of cancer types and is linked to tumor spread and a poor prognosis for patients [29]. Breast cancer cells and the human cervical cell line TCS both overexpressed *LAMP3*, which increased lymph node metastasis in vivo and in vitro, respectively[30]. Furthermore, by triggering regional PKA expression and encouraging VASP phosphorylation at Ser239, the deletion of LAMP3 significantly reduced the motility and spread of esophageal cancer cells[31]. *RTN3* (Reticulon 3) gene is a member of the highly conserved reticulon gene family, which is predominantly expressed in neuroendocrine organs. The activity of beta-amyloid converting enzyme 1 (BACE1) and the creation of amyloid-beta are influenced by interactions between this family of proteins and *BACE1*. The formation of amyloid-beta is markedly decreased when any reticulon protein is expressed more, indicating that reticulon proteins are negative regulators of BACE1 in cells. It was found in a recent study that RTN3 was often downregulated in hepatic cancer cells but robustly expressed in normal hepatocytes. Poor outcome in cancer patients was predicted by low RTN3 expression in a TP53 gene mutation-dependent manner. By activating p53, RTN3 reduced cancer cell development and triggered apoptosis[32]. *BTN2A1* Butyrophilin Subfamily 2 Member A1 gene encodes the immunoglobulin superfamily. The protein that is encoded is a component of the plasma membrane that participates in the metabolism of sterols, fatty acids, and lipids. This gene's variations could be linked to metabolic syndrome and other illness states [33]. It was found that the destruction of tumor cells by V9V Gamma 2 T cells required BTN2A1 expression [34]. A better overall response rate to chemotherapy was also strongly correlated with higher levels of BTN2A1 [35]. *FOXM1* gene, forkhead box M1 produces a protein that acts as a transcriptional activator to promote cell growth. The encoded protein controls the production of multiple cell cycle genes, including cyclin B1 and cyclin D1, and is phosphorylated in the M phase. Few genes are elevated during the early stages of cancer development, including *FOXM1*. Its function in cell cycle progression and proliferation is largely responsible for *FOXM1*'s participation in the start of carcinogenesis. A wide range of tumors, including those of the liver, prostate, brain, breast, lung, colon, pancreas, skin, cervix, ovary, mouth, blood, and nervous system, have elevated expression of *FOXM1*. These results suggest that FOXM1 plays a key role in carcinogenesis. Recent studies have also linked *FOXM1* dysregulation to the development of cancer treatment resistance and the course of the disease[36]. A study has shown that Metformin dramatically reduced FOXM1 protein levels by reducing FOXM1 expression in cancer cells, which resulted in faster apoptosis and cell cycle arrest at the G0/G1 and G2/M phases, both of which reduced cell proliferation [37]. NQO1 gene, NAD (P)H Quinone Dehydrogenase 1 is a quinone from the NAD(P)H dehydrogenase family, producing a 2-electron reductase in the cytoplasm. This FAD-binding protein converts quinones to hydroquinones and creates homodimers. The enzymatic activity of this protein prevents quinones from being reduced by one electron, which would otherwise produce radical species. This gene's mutations have been linked to cancer susceptibility [38]. Recent meta-analysis studies have demonstrated that the replacement of cytosine with thymidine (609C > T) expresses the substitution of serine for proline, reducing the NQO1 enzyme activity, and resulting in the development of several types of human cancers [39]. One study revealed that endometrial tissue treated with metformin showed regression of lesions in the rat model of endometriosis and the potential mechanism could be the overexpression of NQO1 and markers related to autophagy [40]. Standing for the results obtained in our study the NQO1 gene is sensitive to metformin. L1TD1 gene—LINE1 type transposase domain containing 1 predicted to facilitate the action of binding to single-stranded RNA. L1TD1 mRNA expression was found to be frequently downregulated in all of the non-small cell lung cancer cell lines compared to healthy human bronchial epithelial cells. Ectopic expression of L1TD1 also decreased tumor cell viability, proliferation, and the capacity of Non-Small Cell Lung Cancer (NSCLC) cells to form colonies [41].

While the in-silico study using Gene2drug has provided valuable insights into potential gene targets for metformin in cancer treatment, there are several limitations associated with this type of research. In-silico predictions may not always accurately reflect actual biological responses. Experimental validation in laboratory settings, such as cell culture or animal studies, is necessary to confirm the sensitivity of identified genes to metformin and perform functional assays and experiments to assess the impact of metformin on the identified genes and their role in cancer-related processes. In addition, analyses may not fully capture the complex biological context, including tissue-specific variations, microenvironment influences, and interactions with other signaling pathways. The identified genes' sensitivity to metformin can be empirically validated through future laboratory research and preclinical trials. To validate the in-silico predictions, this may entail animal models, cell culture research, and, if relevant, samples collected from patients. Integrating these in-silico findings with existing biological knowledge and conducting additional analyses that consider tissue-specific factors through collaboration with experimental biologists can provide a more comprehensive understanding [42]

## Conclusion

The current study presents a screening protocol following the selection of the candidate's gene using Gene2drug. Nine genes playing a role in cancer were found to be sensitive to metformin. The association between metformin and radio/chemotherapy treatment response in cancer has been demonstrated previously [43]. Although metformin was proven clinically to have some anticancer activities, the mechanism behind this effect is not clearly understood. Hence, this in-silico study holds promise for metformin and gene-targeted treatment strategies in cancer. Here, we conducted *in-silico* screening by Gene2drug. The size limitation of Gene2drug is an important boundary in this study [44, 45]

## Supplementary Information

Below is the link to the electronic supplementary material.

Supplementary file1 (XLSX 28 KB)

Supplementary file2 (XLSX 28 KB)

## Data Availability

This published article and its supplementary information files include all data generated or analyzed during this study.
